# Summary BSR Annual Meeting 2019

**DOI:** 10.5334/jbsr.1959

**Published:** 2019-11-16

**Authors:** Raymond Oyen

**Affiliations:** 1 University Hospitals Leuven, BE

Dear Colleagues,

The 2019 Annual Meeting of the BSR has chosen ‘Pelvic Imaging and Leadership-Management-Quality’ as the main topic. The sections ‘Abdominal Imaging’ and ‘LMQ’ in charge of the organization, in close collaboration with the Young Radiologist Section (YRS), put their largely appreciated efforts in realizing a scientific program with a national and international faculty of experts. Pelvic imaging is indeed a substantial part of the daily practice of many radiologists. Leadership, management and quality issues have become inevitable for departments of radiology and radiologists. Radiologists can no longer passively watch from the side and must take initiatives to be and remain in the driver’s seat.

We take this opportunity to draw the attention to the recently passed International Day of Radiology 2019 (November 8) with ‘Sports Imaging – Musculoskeletal Radiology’ as this year’s main theme. Mainly due to the developments of ultrasound and magnetic resonance imaging, this part of radiology plays an increasingly important role in the assessment and management of patients with a wide variety of injuries, which as much as 70% of people will experience in their lives.

It will be a great pleasure to welcome you in large numbers at the 2019 Annual Meeting of the BSR in Brussels.

Best regards

Prof. Dr. Raymond Oyen

On behalf of the Scientific Council of the BSR

November 2019

